# Effects of Physical Exercise in Nasal Volume

**DOI:** 10.1016/S1808-8694(15)30065-3

**Published:** 2015-10-19

**Authors:** Marconi Teixeira Fonseca, Juliana Altavilla van Petten Machado, Soraya Alves Pereira, Kelerson Moura Pinto, Richard Louis Voegels

**Affiliations:** a PhD in Otorhinolaryngology - FMUSP, Preceptor of Otorhinolaryngology and Head and Facial Surgery - Socor Hospital - Belo Horizonte.; b MD, Intern at the Specialization course of Otorhinolaryngology and Facial Surgery - Socor Hospital - Belo Horizonte.; c MD, Intern at the Specialization course of Otorhinolaryngology and Facial Surgery - Socor Hospital - Belo Horizonte.; d MS in physical Education - Department of Sports Physiology - School of Physical Education - University Center Belo Horizonte.; e PhD, Professor of Otorhinolaryngology, FMUSP Department of Otorhinolaryngology and Head ang Facial Surgery - Socor Hospital - Belo Horizonte; Department of Otorhinolaryngology Medical School of the University of São Paulo (FMUSP). Brazil.

**Keywords:** acoustic rhinometry, nasal volume, physical exercise

## Abstract

The nasal permeability has been demonstrated using several exams. Nasal structures produces a resistance to the nasal air flux that represents over 50% of the total respiratory resistance. Physical exercises is a factor that brings a vasoconstrictor effect over nasal mucosa. **Ains:** Evaluate the improvement degree of nasal volume after aerobic physical exercises and time to return to previous levels. **Subjects and Methods:** Nineteen heathly subjects were submitted to aerobic exercise in ergometric bike. The nasal volume was obtained by Acoustic Rhinometry perfomed in rest, after aerobic exercise, 10o and 20o minutes after the aerobic exercise. **Results:** Rhynometrics results shows a statically and significant increase of nasal volume (p<0,001). The nasal volume, in twenty minutes, returns nearby the rest levels. **Conclusions:** Aerobic exercises, generally, increases the nasal volume. However, the increase of nasal volume was transitory, and occurs a major reduction of increase in the first ten minutes after the exercises ends, and perform a greater vasoconstrictor effect over nasal mucosa, Twenty minutes after the physical exercises finish, total nasal volume returns, closely, to the basal levels.

## INTRODUCTION

Nasal physiology is highly dynamic, it varies according to autonomic mechanisms and also in response to numerous external stimuli. Nasal structures generate a resistance to nasal airflow that may represent 50 to 60% of total respiratory resistance^1^. It is known that many factors such as age, environmental temperature, body posture, medications, hyperventilation, nasal mucosa inflammatory process, hormonal factors, alcohol ingestion and physical exercise may alter nasal resistance^2^.

A number of techniques have been used to measure nasal permeability variation, such as anterior rhinoscopy, MRI, rhinomanometry. They all have their limitations due to cost, discomfort, limited action and poor reproducibility^3^. Acoustic rhinometry (AR) was first described in 1989 by Hilberg et al.^4^ This test assesses nasal volume and it is based on the rebounding of an acoustic wave that brings information about nasal cavity spatial extent. Compared to other methods used to assess nasal permeability, we may say that acoustic rhinometry is fast, reproducible, non-invasive, accurate and of reasonable cost^5^. Since then, acoustic rhinometry excellent reproducibility has been often proven^6^, ^7^.

Nigro et al. (2003) confirmed AR usefulness in the objective evaluation of nasal permeability, especially when changes in nasal geometry are studied in the same individual before and after a certain stimulus^8^.

Studies have shown a significant reduction in nasal resistance during exercise and also a linear relationship between reduction magnitude and load intensity^9^, ^10^. It is also believed that most of the resistance reduction happens right after the beginning of the exercise, and it drops slowly afterwards until 5 minutes following the exercise. The main mechanism responsible for this increase in nasal permeability during physical exercise is discharge from the sympathetic nervous system^11^. A number of factors would be involved in the action of physical exercise on the reduction of nasal resistance: nasal mucosa active vasoconstriction, increase in the activity of the alar nasal muscle, passive blood redistribution for muscles under exercise distant from the nasal mucosa, increase in nasal airflow and hyperventilation^12^.

The goal of the present study was to determine quantitatively the degree of nasal volume improvement with aerobic physical exercise, as well as determining the time nasal volume takes to return to rest levels. Later we will present time and load variations in relation to nasal volume.

## MATERIALS AND METHODS

We selected nineteen healthy volunteers for the study (12 men and 7 women), with ages ranging from 18 to 28 years (average of 22.9 years) for prospective purposes. We excluded those individuals who complained of nasal obstruction, nasal trauma or who had previous nasal surgery, rhinitis, nasal polyps, bronchitis, use of nasal drops or nasal topic steroids, use of contraception medication or beta-blockers and smokers. All volunteers agreed and signed the informed consent form. This study was approved by the Ethics Committee of the Medical School of the University of São Paulo.

The volunteers underwent a general clinical examination, in which respiratory rate, heart rate and blood pressure were assessed. The nose exam comprised external nose inspection, anterior rhinoscopy and nasal fibroscopy.

The selected individuals underwent a “maximum effort test” in order to determine their maximum aerobic capacity, using a mechanical breaking-system stationary Monark® bicycle, according to Balke’s classic protocol. In this protocol, the exercise should start at a power load of 25 watts, with 25 watt load increase every two minutes until fatigue sets in. Fatigue was considered when the individual was no longer able to keep pedaling at a rate of 50 rotations per minute within the established power setting, or when the volunteer said he/she could no longer continue the exercise^13^. The calculation of the individual work power was based on the maximum aerobic power reached during the test.

In order to characterize sample body composition, we carried out sample body mass and height measures using the Welmy® anthropometric scale (marked at 100g and at 1cm). We also measured skin folds, according to Silami-Garcia’s proposal (1990), using the Fitness Scoll software to calculate body fat percentage.

The equipment used to assess nasal volume was the acoustic rhinometer (Eccovision Acoustic Rhinometer - Model AR 1003, Hood Laboratories, Pembroke, MA, USA), which includes a computer with analogue-digital converter, which captures and processes data - there is a module that reproduces acoustic pulse, a tube to transmit the wave to the nostril, a microphone, an amplifier and a filter. A non-invasive nasal adapter was used to connect the sound transporting tube to the nostril under test. In order to avoid sound escaping during the test, water soluble, sterile sealing gel was applied to the adapter border (Lubricating Jelly, Medline Industries, Mundelein, IL, USA), and it was reapplied after each measurement.

According to Fabra and Montserrat Gili, the physical principle of the technique is that the sounds within a tube, in this case the airways, is reflected by changes in acoustic impedance caused by changes in tube dimensions^14^. Changes in the cross-section area are proportional to changes in the acoustic impedance, bearing in mind that sound waves travel in a single dimension. If the striking sound waves are compared to the reflected waves, it is possible to determine changes in the cross-section. All this information is captured by a microphone located in the distal end of the tube, it is amplified and processed by a computer. The time span between the striking and the rebound of the sound waves together with sound velocity determines the altered cross-section area^13^.

For data collection, the volunteer remained seated in the exam room for at least 20 minutes before it began, in order to allow proper acclimatization. The measures were taken according to the Acoustic Rhinometry Standardization Committee of the European Society of Rhinology^15^. For study purposes, we used the measured distance of 6cm proximal to the nasal cavity, and point zero was the nostril opening.

**Figure 1:**
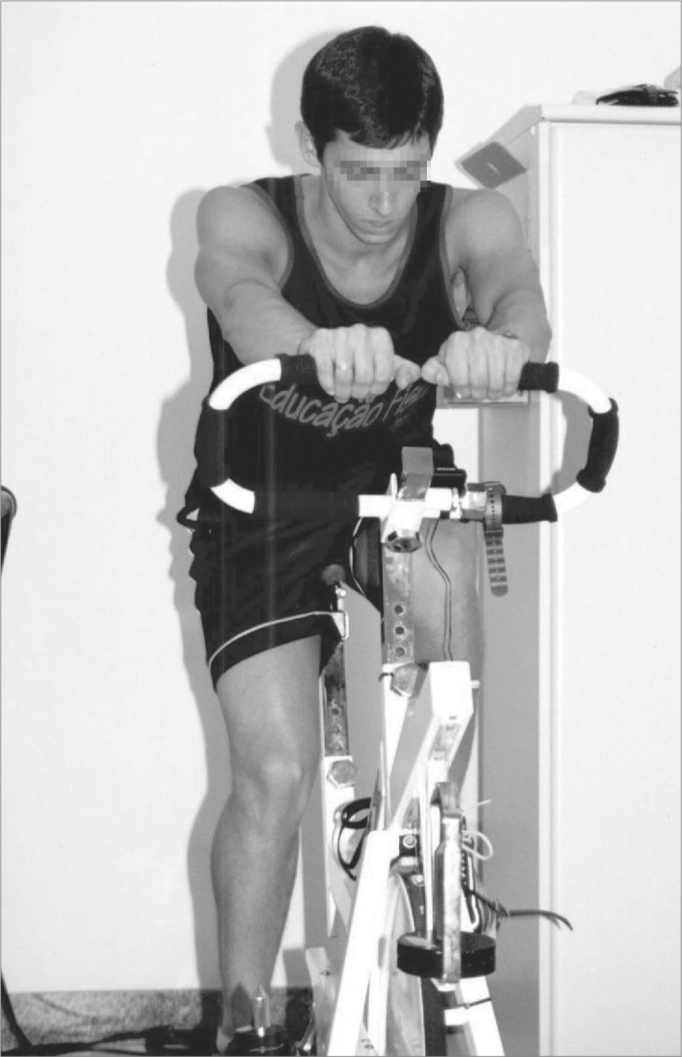

Initially, acoustic rhinometry was carried out in order to determine nasal volume at rest. Following that, the individual exercised for 5 minutes in the stationary bike, at 50% of maximum power load (Figure 1).

Each individual’s nasal volume was obtained through acoustic rhinometry, carried out immediately after the physical exercise and at the 10 and 20 minutes of the end. All rhinometry exams were performed by the same examiner and using the same equipment. In order to avoid external effects that could interfere in result accuracy, each individual had his/her data collected in one day. All tests were carried out in the same room and within the same season of the year (spring). The temperature of the exam room was measured through a mercury thermometer, in such a way that we avoided to carry out the test when room temperature varied in more than 2.5°C in relation to other days in which the tests had been carried out.

Nasal volume measure (NV) was obtained for each nasal cavity separately. The volumes of both nasal cavities were added up, thus obtaining the Total Nasal Volume (TNV). For each individual we carried out a comparative calculation between the nasal value obtained and the values obtained after the test. This calculation was carried out in percentages, having 100% as baseline value. We used the Friedman Test for statistical analysis, considering 95% for significance level (p< 0.05).

## RESULTS

Room temperature measured in different days of data collection presented a maximum value of 28.5°C and a minimum value of 26°C, with an average of 27.06°C. Average humidity in Belo Horizonte at the time (September to December of 2002) was of 68.15%, according to the 5th District of Meteorology / INMET.

In the 19 volunteers studied, the lowest TNV found at rest was 10cm^3^ and the highest TNV found was of 16.55cm^3^, the average TNV observed was 12.1cm^3^ (± 1.7).

**Graph 1:**
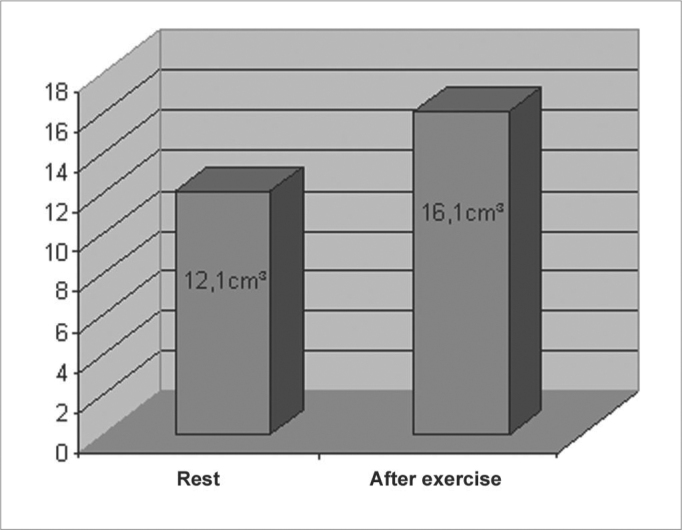

The TNV increase was of 33.3% (± 17.0) in relation to rest values (Graph 1). The significance index (p) was lower than 0.001.

**Graph 2:**
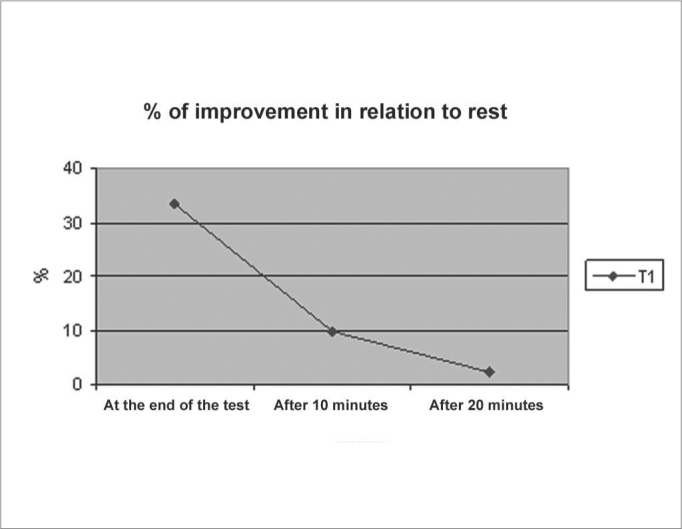

We could see that the average values for nasal volume went up at the end of each test, and later they dropped gradually. The statistical comparison shows high significance in this alteration (rise and then fall). As we can see on Graph 2, in the 20th minute the averages of nasal volumes were very close to the value at rest. In the 20th minute we found TNV: 12.4cm^3^ (± 1.6) (Graph 2).

## DISCUSSION

The increase in nasal permeability accruing from physical exercise is a data that has been proven in the literature. This increase is mainly a consequence of vascular alterations that happen in the lateral nasal wall, through mucosa decongestion, producing a reduction in nasal resistance, allowing an increase in nasal air volume that aims at preparing it as best as possible to supply that new demand for air^16^. This vascular tissue occupies almost all the lateral nasal wall, and its largest concentration is in the anterior half of the inferior conchae. The vascular tissue that responds to vasoconstriction starts at 2 cm within the nose and goes until approximately 6cm^17^. In physiological terms, the Respiratory is one of the systems that most suffers alterations when a person is exercising. Within this system, it is the nose that offers the greatest resistance to nasal airflow, and it is right there we observe the most effects brought about by physical exercises^1^.

In our study we tried to evaluate the effects of physical exercise over the nose through the assessment of nasal volume alterations, what best translates the degree o decongestion of nasal mucosa. Within the aforementioned observations, we have calibrated our system to measure the first 6 centimeters of the nasal cavity. Thus, we would be measuring the area within the nasal cavity that bears the highest concentration of responsive erectile tissue to physical exercise. We would also be avoiding the choanal region, where most air escapes and this could impair the measurement accuracy^15^.

In selecting the individuals who would participate in the study, we did not separate them by gender, because it has been established that aerobic capacity does not bear differences as far as gender is concerned^18^. As to age, we know that the aerobic capacity (VO2max) hits its peak at 18 years of age, and it is kept constant until 30 years of life^17^. According to this assumption we had volunteers within this age range, the youngest was 18 years of age and the eldest 28, the group averaged 22.9 years. We tried to selected individuals who were not obese, because obese persons have VO_2_max values lower than what is expected^18^. In the group studied, body fat index (BFI) presented amplitudes varying from 9.51% to 28.94%, with an average value of 18.08%. Finally we did not take into account if the volunteers practiced any regular physical activity, since nasal resistance at rest does not bear differences when we compare athletes to sedentary individuals^2^. From the stand point of acoustic rhinometry, age, gender, height, weight and body mass factors did not influence their values^19^.

Most studies about the effects of physical exercise over nasal permeability used other evaluation methods such as rhinomanometry or pletismography^9^, ^10^, ^11^, ^12^. Because of its advantages in relation to other objective evaluation methods to assess nasal permeability, we decided to use acoustic rhinometry to obtain our data. During data collection we tried, besides following the precepts established for the exam performance^15^, to avoid external factors that could interfere in its accuracy, such as temperature variations above 2.5° C between exam days^20^, at least 20 minutes acclimatizing before the exam was carried out^21^, keeping a low noise environment and have the exam performed by the same trained professional^22^.

In regards to the goals of this study, we found that our results are in agreement with other studies, that is, physical exercise does cause a significant increase in nasal permeability^9^, ^11^, ^12^. Forsyth et al.^10^ and Lacroix et al.^16^ used nasal resistance as assessment parameters and could see, respectively, a reduction of 30 to 46% and of 31% after physical exercise. We saw a nasal volume increase of 33.3% in relation to rest values.

According to the literature, the effect of physical exercises over nasal permeability is transitory and its return to baseline levels happen within 30 minutes of its end^10^, ^12^, ^16^. In our study we could see that at the 20th minute the nasal volume had returned to its rest level. We could also see two other things. First: the reduction in improvement level in TNV was more intense in the first 10 minutes after the exercise. Between the 10th and the 20th minute the reduction level was less significant.

## CONCLUSION

This study confirmed the finding of other studies that isotonic physical exercises cause a statistically significant increase in nasal volume. The increase in nasal volume was transitory, a greater reduction in this increase happened in the first ten minutes after the end of the exercise, and 20 minutes after the end of the exercise nasal volume values fell close to those found at rest.
